# Development of Seven Microsatellite Markers Using Next Generation Sequencing for the Conservation on the Korean Population of *Dorcus hopei* (E. Saunders, 1854) (Coleoptera, Lucanidae)

**DOI:** 10.3390/ijms160921330

**Published:** 2015-09-07

**Authors:** Tae Hwa Kang, Sang Hoon Han, Sun Jae Park

**Affiliations:** 1 Plant Quarantine Technology Center, Animal and Plant Quarantine Agency, 234-3, Mangpo-dong, Yeongtong-gu, Suwon, Gyeonggi-do 443-400, Korea; 2 Department of Life Science, College of Natural Science, Kyonggi University, 154-42, Gwanggyeongsan-ro, Yeongtong-gu, Suwon, Gyeonggi-do 443-780, Korea; 3 Biological Resources Research Department, National Institute of Biological Resources, 42, Hwangyeong-ro, Seo-gu, Incheon 404-170, Korea

**Keywords:** Coleoptera, *Dorcus hopei*, hybrid population, Lucanidae, microsatellite marker, MiSeq, next generation sequencing, pet insect

## Abstract

We developed microsatellite markers for genetic structural analyses of *Dorcus hopei*, a stag beetle species, using next generation sequencing and polymerase chain reaction (PCR)-based genotyping for regional populations. A total of 407,070,351 base pairs of genomic DNA containing >4000 microsatellite loci except AT repeats were sequenced. From 76 loci selected for primer design, 27 were polymorphic. Of these 27 markers, 10 were tested on three regional populations: two Chinese (Shichuan and Guangxi) and one Korean (Wanju). Three markers were excluded due to inconsistent amplification, genotyping errors, and Hardy-Weinberg equilibrium (HWE). By multi-locus genotyping, the allele number, observed heterozygosity and polymorphism information content of seven microsatellite loci were ranged 2‒10, 0.1333‒1.0000, and 0.1228‒0.8509, respectively. In an analysis on the genetic differentiation among regional populations including one Japanese population and one cross-breeding population, the individual colored bar-plots showed that both Chinese populations were closer to each other than to the Far East Asian populations. In Far East Asian populations, Wanju and Nirasaki populations could not be distinguished from each other because the frequency of genetic contents was very similar in some individuals of two populations. Moreover, the cross-breeding population contained all patterns of genetic contents shown in Chinese, Korean, and Japanese populations, compared with the genetic content frequency of each regional population. As a result, we examined whether the cross-breeding population might be a hybrid population, and might contain a possibility of interbreeding with Chinese populations in parental generations. Therefore, these markers will be useful for analyses of genetic diversity in populations, genetic relationships between regional populations, genetic structure analyses, and origin tests.

## 1. Introduction

*Dorcus hopei* [1] (Lucanidae, Coleoptera), belonging to the group of stag beetles, is widely distributed throughout China and Japan. The species is divided into two subspecies, *D. hopei hopei*, which is distributed throughout central China, and *D. hopei binodulosus* [2], which is found in northeastern China, Korea, and Japan [3,4]. *D. hopei* can be distinguished from other closely-related species by its mandible shape (thick mandible with one inner tooth) in males, and the elyral puncture line in females. *D. hopei* is one of the largest Coleoptera species in East Asia: males can grow up to 76 mm in the wild [3,4,5]. However, it was recently reported to be a rare species in Korea and Japan [3,6].

The market for insects as pets has increased due to growing interest in keeping exotic pet animals. The stag beetle market is worth nearly $283 million in Japan, while the market in Korea is estimated to be worth approximately $38 million [7,8]. Some insect collectors called *D. hopei binodulosus* “Black Diamond” because its body color was black and as expensive as diamonds [6,9,10]. Because of their worth, many stag beetle collectors have tried to find large specimens of *D. hopei* and some insect collectors have tried to cross-breed the insect with different regional populations because they believed that the method could increase the body size of the insect [11,12]. This has led to an influx of foreign populations and cross-breeds into the wild by insect collectors who lost interest in breeding them.

In Japan, this insect has been popular as a pet for more than 30 years, and large-sized individuals (>80 mm) have been traded at high prices [6,9,10]. This has led to negative effects on *D. hopei* populations in Japan and Korea, including indiscreet collecting and habitat destruction. Thus, in Japan the species was listed up in the Japanese Red Lists [13], and in Korea it was recommended to be treated as a “Preserved Wild Animal II” [3,14]. More recently, the Animal and Plant Quarantine Agency of Korea created the “Import Regulations on Pet and Natural Enemy Insects” in March of 2013 [15]. It was considered that these invasions were not limited to local areas but that they were linked to whole distribution ranges of the species.

In Korea, there is a thriving trade in foreign beetle populations for cross-breeding. This influx of foreign populations combined with indiscriminate collections of the insect could result in allele frequency change in native Korean populations. This fact caused several requests for developing a monitoring tool to investigate a change genetic diversity of *D. hopei* in Korea. For this reason, we decided to analyze the genetic diversity among Chinese, Korean, and Japanese populations of *D. hopei*.

Analyses of genetic diversity using microsatellite loci have been carried out for various eukaryotes [16,17,18]. More recently, they have been used to track the influx of invasive species [19,20,21,22,23,24]. However, a primer set for each polymorphic locus is needed in the use of microsatellite loci. The general method employed is an enrichment strategy [25,26], which is expensive and time-consuming, as it is based on the traditional cloning strategies [27,28,29]. In this study, Illumina sequencing, one of next generation sequencing (NGS) techniques, was used to read genomic DNA of *D. hopei*, and then microsatellite markers were developed from the results. NGS technique is very useful for the construction of microsatellite loci libraries at a lower cost and far more quickly than traditional cloning-based approaches [20,28,30]. The aim of this study was to develop and test microsatellite markers for analyses of population structure, relatedness, and genetic diversity in *D. hopei* populations.

## 2. Results and Discussion

### 2.1. Microsatellite Marker Development

By Illumina sequencing of genomic DNA, we generated 407,070,351 base pairs from 29,383,396 reads, with an average of 248 base pairs (Table 1). Then the 29,383,396 reads were assembled into a draft genome, which contained approximately 4000 microsatellite loci (excluding AT repeats). Of these, 76 loci, composed of di- to hexa-nucleotide repeats, were selected for primer design and polymerase chain reaction (PCR) (Table S1). By polymerase chain reaction (PCR) analysis, 48 loci showed polymorphic amplification; among these, 12 loci showed ambiguous amplification and nine primer sets did not produce any visible amplicon from some regional populations. The remaining 27 primer sets were deemed as candidate loci for use in genetic diversity and relatedness analyses for each of the regional populations (Table S1).

**Table 1:** Summary on next generation sequencing result of *Dorcus hopei.*

Sequence Type	Number of Obtained Sequences	Average Length of Obtained Sequences	Total Base Pairs
Reads	29,383,396	247.63	7,276,297,814
Contigs	334,033	1218	407,070,351

### 2.2. Microsatellite Marker Assessment

We randomly selected 10 of the 27 primer sets, and tested for marker availability on 53 samples of three regional *D. hopei* populations (16 samples from Shichuan (China), 17 from Guangxi (China), 20 from Wanju (Korea)). However, one marker (33136) did not show stable amplification and could not be tested. Nine markers amplified DNA fragments containing allele numbers ranging from 2 to 14 in 53 stag beetles. The linkage disequilibrium analysis on each regional population was conducted. From the result, we did not detected linkage disequilibrium across loci over all samples. Then, we tested the present of null allele (Table 2). Five microsatellite loci showed null allele evidence: Locus 10203, 271200, 516192, 638128, and 668183. Among them, four loci, 10203, 271200, 516192, and 668183, showed the frequency of null allele of *p* < 0.20, but locus 638128 as *p* > 0.20. When microsatellite null alleles are uncommon to rare (*p* < 0.20), their presence causes a slight underestimate of the average exclusion probability at a locus, but probably not of sufficient magnitude to warrant great concern, but, in *p* > 0.20, mean “estimated with null” exclusion probabilities can be much higher than the “true” and “estimated without null” values [31]. Thus, above four loci showing as *p* < 0.20 could be used for population genetic analysis in this study, but 638128 was excluded.

**Table 2:** The frequency of null alleles on each regional population.

Microsatellite Locus	Shichuan		Guangxi		Wanju	
	Null Present	Frequency	Null Present	Frequency	Null Present	Frequency
10203	no	0.0593	no	0.0258	yes	0.1925
76181	no	−0.7500	no	−1.0000	no	−1.0000
271200	no	−0.0885	yes	0.1669	no	−0.2152
516192	no	0.0165	no	0.0617	yes	0.1965
585164	no	0.0120	no	0.0165	no	−0.1411
638128	yes	0.3169	no	0.1889	yes	0.2672
668183	no	−0.0023	yes	0.1979	no	0.0706
951171	no	−0.0678	no	−0.0925	no	0.0927
1539174	no	0.0070	no	0.0252	no	0.0277

For each marker, gene diversity, observed heterozygosity, and the polymorphism information content (PIC) ranged 0.1267‒0.8651, 0.1333‒1.0000, and 0.1228‒0.8509, respectively. Gene diversity and the PIC were highest in locus 10203, while heterozygosity was highest in locus 76181. Locus 951171 had the lowest values of all three indices (Table 3). For the regional populations, gene diversity was highest in the Shichuan population, with a mean value of 0.7057, and lowest in the Wanju population, with a mean value of 0.6448. The Shichuan population was the most heterozygous, with a mean value of 0.7655, and the Guangxi population was the least heterozygous, with a mean value of 0.6567. The Shichuan population had the highest PIC mean value of 0.6619, while the Wanju population had the lowest PIC mean value of 0.5912 (Table 3). HWE were estimated after sequential Bonferroni corrections (*p* = 0.002). Through exact *p*-values of HWE on each locus, we examined that locus 76181 was out of HWE in all regional populations tested for marker assessment (Table 3). Thus, the locus was excluded from our population structure analysis of the stag beetles. In Wanju population, exact *p*-value of each locus was relatively low except locus 1539174 showing 0.7140. Over-collecting for the increasing collectible value of *D. hopei binodulosus* in the Korean breeders might come to this result. In the collecting information on Wanju regional population, eight samples were collected at 2008 and 12 samples at 2015, respectively (Table S2). The stag beetle was distributed in whole of Korean Peninsula [4], but their habitats were decreased during recent years [3]. Therefore, we considered that the result of HWE on Wanju regional population might be caused by decreasing of genetic diversity due to over-collecting of the Korean breeders.

**Table 3:** Characteristics of eight polymorphic microsatellites loci developed in three regional populations of *Dorcus hopei* (MSL, Microsatellite locus; RP, Regional population; MAF, Major allele frequency; Gn, Genotype number; SS, Sample size; An, Allele number; GD, Gene diversity; Ho, Observed heterozygosity; PIC, Polymorphism information content; HWE, Exact *p*-value of Hardy-Weinberg Equilibrium).

MSL	RP	MAF	Gn	SS	An	GD	Ho	PIC	HWE
10203	Guangxi	0.2059	12.0000	17.0000	10.0000	0.8651	0.8235	0.8509	0.0170
	Shichuan	0.2188	15.0000	16.0000	9.0000	0.8496	0.7500	0.8317	0.5860
	Wanju	0.2750	14.0000	20.0000	9.0000	0.8138	0.5000	0.7897	0.0020
76181	Guangxi	0.5000	1.0000	17.0000	2.0000	0.5000	1.0000	0.3750	0.0000
	Shichuan	0.5313	2.0000	16.0000	2.0000	0.4980	0.9375	0.3740	0.0000
	Wanju	0.5000	1.0000	20.0000	2.0000	0.5000	1.0000	0.3750	0.0000
271200	Guangxi	0.4412	10.0000	17.0000	8.0000	0.7007	0.4706	0.6567	0.0630
	Shichuan	0.4063	13.0000	16.0000	9.0000	0.7813	0.8750	0.7612	0.9940
	Wanju	0.4750	6.0000	20.0000	5.0000	0.6663	0.9000	0.6140	0.0080
516192	Guangxi	0.3529	11.0000	17.0000	7.0000	0.7543	0.6471	0.7175	0.2910
	Shichuan	0.4063	11.0000	16.0000	8.0000	0.7520	0.7500	0.7204	0.5460
	Wanju	0.6750	4.0000	20.0000	3.0000	0.4663	0.2500	0.3947	0.0050
585164	Guangxi	0.5882	6.0000	17.0000	5.0000	0.5467	0.5294	0.4719	0.8820
	Shichuan	0.5938	6.0000	16.0000	5.0000	0.5625	0.5625	0.5017	0.4280
	Wanju	0.3750	8.0000	20.0000	7.0000	0.7188	0.9000	0.6691	0.0650
668183	Guangxi	0.5588	9.0000	17.0000	9.0000	0.6522	0.4118	0.6293	0.0010
	Shichuan	0.4333	9.0000	16.0000	7.0000	0.7311	0.7333	0.6965	0.5200
	Wanju	0.3500	7.0000	20.0000	4.0000	0.7238	0.6000	0.6723	0.0000
951171	Guangxi	0.9118	2.0000	17.0000	2.0000	0.1609	0.1765	0.1480	1.0000
	Shichuan	0.9333	3.0000	16.0000	3.0000	0.1267	0.1333	0.1228	1.0000
	Wanju	0.8500	3.0000	20.0000	3.0000	0.2650	0.2000	0.2469	0.0620
1539174	Guangxi	0.3750	13.0000	17.0000	9.0000	0.8008	0.7500	0.7815	0.5340
	Shichuan	0.4375	12.0000	16.0000	10.0000	0.7656	0.7500	0.7477	0.5650
	Wanju	0.5500	11.0000	20.0000	7.0000	0.6525	0.6000	0.6239	0.7140
Mean	Guangxi	0.5179	7.6667	17.0000	6.2222	0.6025	0.5646	0.5602	-
	Shichuan	0.4921	8.6667	16.0000	6.7778	0.6409	0.6380	0.6022	
	Wanju	0.5333	6.6667	20.0000	5.0000	0.5801	0.5667	0.5306	

### 2.3. Genetic Differentiation among Regional Populations

**Figure 1:**
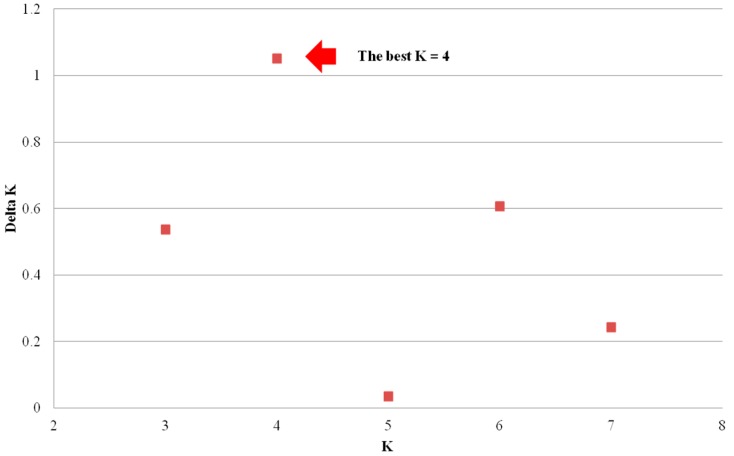
The *ad-hoc* statistics ∆(*K*) on the basis of LnP(D) estimated 20 iterations for each *K*. The *ad-hoc* statistics exhibited a signal at best *K* = 4 (the red arrow).

For analysis on genetic differentiation among regional populations, 37 samples from Nonsan, Korea and 6 samples from Nirasaki, Japan were included in this study based on a model-based Bayesian analysis. *K* was estimated with varying from one to 10, and the *ad-hoc* statistics Δ(*K*) [32] indicate an uppermost level of structure in four populations (Figure 1).

**Figure 2:**
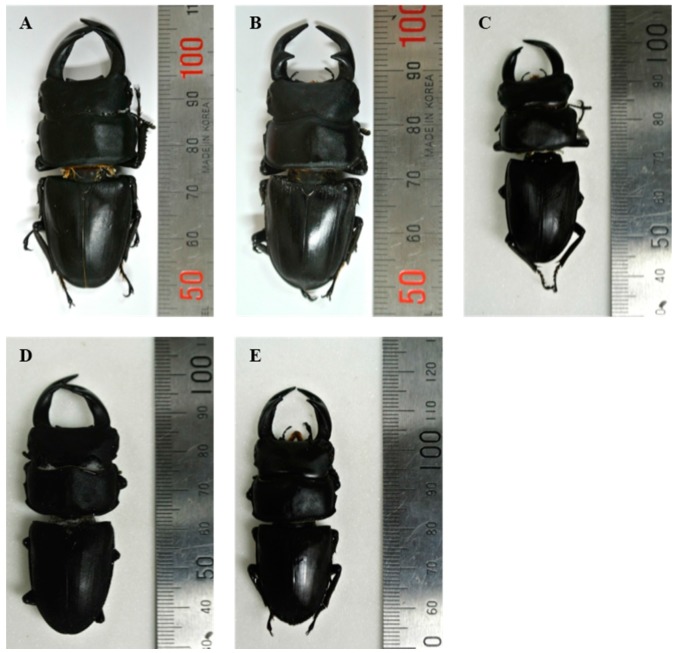
Comparsion of body length on each regional population of *Dorcus hopei*. (**A**, Shichuan population, about 58 mm; **B**, Guangxi population, about 50 mm; **C**, Wanju population, about 58 mm; **D**, Nonsan population (cross-breed), about 70 mm; **E**, Nirasaki population, about 60 mm).

**Figure 3:**
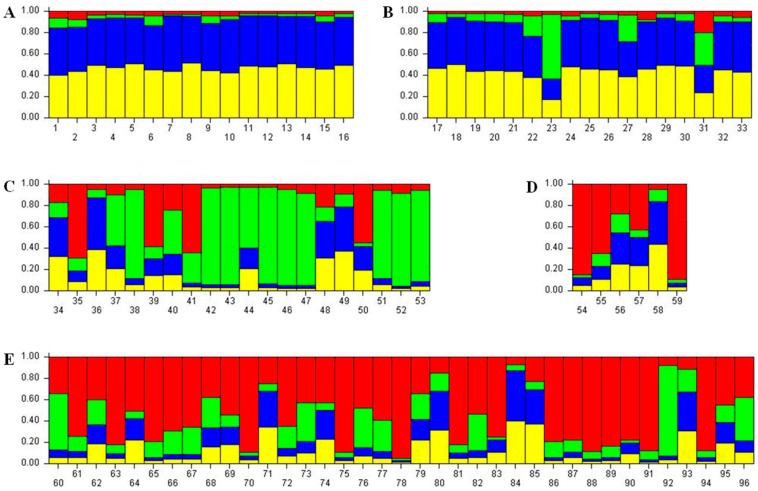
The bar plots in multiple lines were estimated by STRUCTURE. The best *K* was estimated as four based on the *ad-hoc* statistics ∆(*K*) (**A**, Shichuan China; **B**, Guangxi China; **C**, Wanju Korea; **D**, Nirasaki Japan; **E**, Nonsan Korea provided from insect breeder).

**Figure 4:**
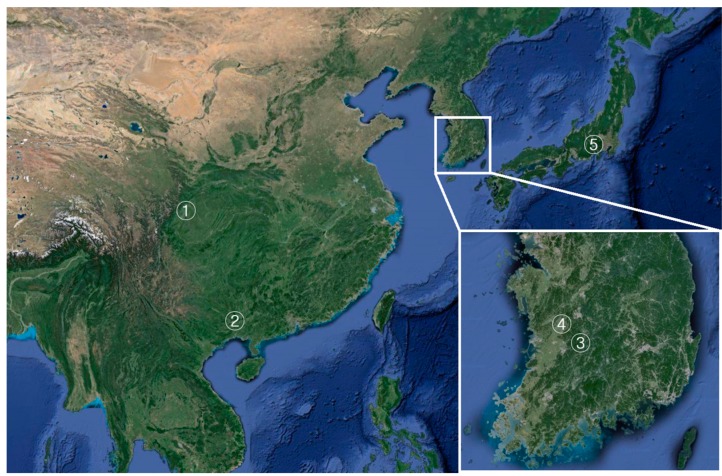
Collecting sites on each regional population of *D. hopei* (1, Shichuan China; 2, Guangxi China; 3, Wanju Korea; 4, Nonsan Korea; 5, Nirasaki-shi Japan).

Total five populations (two Chinese population, Shichuan and Guangxi; two Korean population, Wanju and Nonsan; one Japanese population, Nirasaki) were used for a model-based Bayesian analysis. Among them, Nonsan population was provided from a Korean insect breeder. Interestingly, a specimen of Nonsan population showed the biggest body size among tested samples of this study (Figure 2). Comparing with the colored individual bar plots of each population, the frequency of genetic contents was differed from each region (Figure 3). Chinese populations (Shichuan and Guangxi) and Far East Asian populations (Wanju and Nonsan of Korea, Nirasaki of Japan) appeared clear difference in the frequency of the genetic contents reflecting subspecific difference and geographical distance (Figure 4). Chinese populations showed high frequency on yellow and blue color in genetic contents, but Far East Asian populations were differed in each individual (red, green, or blue color of genetic contents). In Chinese populations, the frequency of each genetic content of Guangxi population was very similar to Shichuan population (Figure 3A,B). In Far East Asian populations, Wanju population of Korea showed that the frequency of genetic contents of each individual was variable in the samples collected in 2008 (Figure 3C: individual no., 34–41) while the frequency of genetic content of green color was high in the samples collected at 2015 (Figure 3C: individual no., 42–53). Moreover, four individuals (Figure 3C: individual no., 34, 36, 48, 49) were very similar to two Chinese populations in the pattern of genetic contents. Although a sample size of the Nirasaki population of Japan was small as only six individuals were studied, we could examine that in the Nirasaki population of Japan, the red color was a major genetic content, and the population contained the individual (Figure 3D: individual no., 58) which might be originated from China (Figure 3D). We could not distinguish the Wanju population from Nirasaki because the frequency of genetic contents was very similar in several individuals of two populations. The Nonsan population was purchased from an insect breeder of the region. According to the statement of the breeder, those beetles were offspring of cross-breeds. The breeder insisted that they were cross-breeds between Korean and Japanese *D. hopei*. However, compared with the genetic content frequency of each regional population, the Nonsan population contained all patterns of genetic contents shown in Chinese, Korean, and Japanese populations (Figure 3E). As a result, we proposed that the Nonsan population might be a hybrid population as breeder stated, but the population contained a possibility of interbreeding with Chinese populations in parental generations. Therefore, the markers developed in this study would still be useful for identifying genetic diversity and relatedness between regional populations of *D. hopei*.

## 3. Experimental Section

### 3.1. Sample Preparation and Genomic DNA Extraction

We used a total of 97 specimens, including 16 from Shichuan, China, 17 from Guangxi, China, 21 from Wanju, Korea, 37 from Nonsan, Korea, and 6 from Nirasaki, Japan (Table S2 and Figure 4). Nonsan specimens were purchased from an insect breeder of the region. Collecting on wild specimens of Korea was conducted in the known distributional regions of Korean peninsula [3], but the wild individuals were captured from only one area (Wanju). Thoracic muscles of all collected samples were removed for genomic DNA isolation, and the samples were dried before being sent to the National Institute of Biological Resources (Incheon, Korea). Genomic DNA for PCR and genotyping was isolated from the thoracic muscle using a DNA purification kit (PrimePrep Genomic DNA Isolation Kit; GeNet Bio, Daejeon, Korea), according to the manufacturer’s instructions.

### 3.2. Illumina Sequencing, Microsatellite Marker Selection, and Genotyping

Genomic DNA for Illumina sequencing was extracted from the thoracic muscle of samples collected from Wanju (Table S2), Korea, using NucleoSpin^®^ Tissue Kit (Macherey-Nagel GmbH and Co. KG, Düren, Germany). DNA quality was checked by electrophoresis on a 1% agarose gel and by spectrophotometry, after which 10 μg of genomic DNA was used for 2 × 300 paired-end sequencing of MiSeq Sequencer (Illumina, San Diego, CA, USA). The contigs obtained via Illumina sequencing were assembled to make a partial draft genome using the CLC Genomics Workbench (Qiagen, Hilden, Germany).

Phobos ver. 3.3.12 [33], a tandem repeat search tool, was used to search for microsatellite loci in the assembled partial draft genome. The searching criteria were 10% mismatch di-, tri-, tetra-, penta-, and hexa-nucleotide repeats with a minimum of 20 base pairs. AT repeats were excluded from the screening result. All primer sets which amplify microsatellite loci were designed by using Primer3 ver. 0.4.0 [34,35] according to the following criteria: GC content >30%, final product length 164–296 base pairs, primer length 18–27 base pairs, and optimal annealing temperature of 56–61 °C (the remaining parameters were at the default settings). 76 primer sets selected and tested for specificity and the presence of polymorphic amplification by PCR with five samples from each regional population (Table S1). PCR for primer qualification test was conducted with AccuPower PCR PreMix (Bioneer, Daejeon, Korea) in a final volume of 20 μL containing 30 ng of template DNA, 5 pmol of each primer. Extra MgCl_2_ was not added. The amplification profile was as follows: 5 min at 94 °C; 35 cycles of 20 s at 94 °C, 20 s at 60 °C, and 30 s at 72 °C; with a final 7-min extension at 72 °C.

Each forward (sense) primer for genotyping was labeled with 6-carboxyflouorescein at the 5′ end [36]. An availability test for markers was carried out on a total of 96 samples. To reduce polymerization error, high fidelity polymerase was used to PCR for genotyping which was carried out in a final volume of 25 μL and contained 30 ng of template DNA, 0.5 U of FR-Taq DNA polymerase (Bio Basic Inc, Markham, ON, Canada), 1× PCR buffer, 200 μM dNTPs, and 5 pmol of each primer. Extra MgCl_2_ was not added. The amplification profile was as follows: 5 min at 95 °C; 35 cycles of 30 s at 95 °C, 30 s at 50–55 °C, and 1 min at 72 °C; with a final 7-min extension at 72 °C. Amplicons from each sample were genotyped using a 3730XL DNA analyzer (Applied Biosystems, Carlsbad, CA, USA). The sequences on the microsatellite loci of the developed markers were submitted to NCBI Genbank and provided with Genbank assession number (Table S1).

### 3.3. Data Analysis

The summary statistics and Hardy-Weinberg equilibrium of each locus were estimated using PowerMarker ver. 3.5 [37]. We ascertained the allele type frequencies based on microsatellite loci for each population (Table S3). The pairwise linkage disequilibrium on the microsatellite loci was carried out with Arlequin ver 3.1 [38] and null allele test was conducted with Micro-Checker [39]. We also tested the genetic differentiation among regional populations based on a model-based Bayesian analysis using STRUCTURE ver 2.3.4 [40,41] (First step: a correlated-allele model with 10,000 of burnin period and 100,000 of MCMC reps after burnin; set *K* from 1 to 10; number of iterations 20, Second step: a correlated-allele model with 500,000 of burnin period and 750,000 of MCMC reps after burnin; set *K* from 2 to 8; Number of iterations 20). Then, ∆(*K*) was estimated with average of LnP(D). The *ad-hoc* statistics ∆(*K*) [32] was used for estimating the number of populations.

## 4. Conclusions

In this study, we used NGS to develop microsatellite markers, which is more cost-effective and quicker to construct a genomic DNA library using NGS than implementing traditional library-based strategies [25]. We tested 76 microsatellite loci for *D. hopei* which had no genomic DNA information available. According to PCR efficiency tests, 27 showed polymorphic patterns in electrophoresis analyses. 10 of 27 markers were randomly selected for genotyping and results from seven markers were used on the final analysis.

As a result, we designed new species-specific microsatellite markers that could provide the basis for future conservation projects with *D. hopei*. Notably, relatively few studies exist on the population genetics of *D. hopei*, despite the high market value of the species as a pet in Korea and Japan. The microsatellite markers could be used for population structure analyses.

## Supplementary Materials

Supplementary materials can be found at http://www.mdpi.com/1422-0067/16/09/21330/s1.
