# Supplementary material for: Development of Seven Microsatellite Markers Using Next Generation Sequencing for the Conservation on the Korean Population of Dorcus hopei (E. Saunders, 1854) (Coleoptera, Lucanidae)
Source: Int J Mol Sci. 2015 Sep 7;16(9):21330–41. doi: 10.3390/ijms160921330 (PMC4613255; doi:10.3390/ijms160921330)
Supplement: Supplementary file 1 [file ijms-16-21330-s001.pdf]

Supplementary Information

Table S1. Primer sets for selected 76 microsatellite loci (UP: unambiguous and polymorphic locus, SL: selected locus for marker optimization, GAn: Genbank accession number).

No.	Locus	Primer Sequence	Repeated Motif	Sequence Size	Remarks
1	10203F<br>10203R	GCGTTGAGCACTGTACCTGA<br>TTCAAACTGAGTGGTACCGTAAAA	$(TG)_{13}$	227	UP, SL<br>GAn: KT591166
2	33136F<br>33136R	GGCAGTAGTGGGAAATTGTCA<br>GCCTGCCACATAAAAGTCCT	$(CAT)_{8}$	225	UP, SL<br>GAn: KT591167
3	63105F<br>63105R	CTCACGAGCCTTCACATTCA<br>AATTGTCGTTTTTCCGTTCG	$(GT)_{12}$	225	UP
4	76181F<br>76181R	AGCACGTTTTGTCCCACTTG<br>CAGAAGCTCTACTGGGGTACG	$(CT)_{10}$	218	UP, SL<br>GAn: KT591168
5	125190F<br>125190R	ACGATCCTACTCCGGCTTTA<br>CGTCGCGTTATGAGCTAGAAA	$(CCGTG)_{4}$	222	UP
6	204211F<br>204211R	CTGCGGAGGACGTGATTATT<br>GGCAAAGGTCTGAGATGGAC	$(CTAT)_{16}$	267	-
7	205168F<br>205168R	GTGCTGCGGAGGACAATC<br>GGTGGCAAAGGTTTGAGATG	$(CTAT)_{10}$	194	-
8	271200F<br>271200R	AAATGCCCAAGAGCTCGTTAT<br>GCCGCCTGCTTCTGTAAAC	$(CA)_{13}$	226	UP, SL<br>GAn: KT591169
9	303184F<br>303184R	CACGGAACGGTTACCAGAAG<br>GGGCAAACCTGGAAGTTGT	$(TGC)_{7}$	221	-
10	326182F<br>326182R	GCGCGCAGACGTGTTAGTA<br>CAAAGAAATCGAAGAGGGTCTC	$(TTG)_{7}$	222	-
11	401155F<br>401155R	GGGTACAAGTTGAAGCGATTT<br>TCTGCTGCACGATCTGTATG	$(ACG)_{7}$	223	-
12	401187F<br>401187R	CCCGGACAGCACAGATTAAA<br>TCTTTCAAGCTAACCGTGCAT	$(TGAATG)_{5}$	230	-
13	516192F<br>516192R	AAATGACCAAATCCGAAAGG<br>TTTTACGGCGGCTGTATCAC	$(ACC)_{7}$	221	UP, SL<br>GAn: KT591170
14	538199F<br>538199R	CTGCGGAGGACGTGATTATT<br>GGCAAAGGTTTGAGATGGAC	$(CTAT)_{13}$	240	-
15	585164F<br>585164R	AATTCACGAGGCTGCATTTT<br>CACGGATACGTAGGGAGTACG	$(AG)_{14}$	164	UP, SL<br>GAn: KT591171
16	591177F<br>591177R	CTTGAAGGCCATTGGGAAAT<br>TCCCCACAGTATTCCAGCAT	$(TC)_{12}$	225	-
17	638128F<br>638128R	ACGTTTTCGTTTGTCCGTTG<br>GATTTTCCCCGCAAAACTTC	$(CGA)_{7}$	219	UP, SL<br>GAn: KT591172
18	652240F<br>652240R	ACCTGGCGAACCTGATACAC<br>CAAAAGAACACAAACGTCTCG	$(GAGGAA)_{9}$	296	-
19	668183F<br>668183R	TGCATTGTGTTTTATTTTTATCAGC<br>TCGATATTCCAATCCAAACCA	$(TGA)_{9}$	228	UP, SL<br>GAn: KT591173
20	686148F<br>686148R	ATCCAGGCTAGTGCCAGAAC<br>GAGAAGGGCTAACTCGCTCA	$(CAGA)_{5}$	188	-

Table S1. *Cont.*

No.	Locus	Primer Sequence	Repeated Motif	Sequence Size	Remarks
21	752140F<br>752140R	CCATTCTCCAATCAAGGAAAA<br>CAGAAATTTGCAAGGGGTCA	$(CAGCT)_7$	237	-
22	807181F<br>807181R	ACCAAGAGAAGCGAGACCAA<br>TAGCAGTTGATGCCGTTTGA	$(GCC)_7$	219	-
23	809183F<br>809183R	AGCCAGCTGAAGGAACACAC<br>ACGACGTTTCGAATCCACTC	$(TCCCAT)_4$	228	UP
24	814124F<br>814124R	TTGTTTTCAGCTGTCGAGGA<br>CCCGGCTTTCAGGTATTACA	$(TGT)_7$	221	-
25	853148F<br>853148R	AGTTTGCACGCCCTCTATTG<br>TTGCACACTCACGTTCTTCC	$(TGTGT)_4$	222	-
26	914143F<br>914143R	TTGCCAAAAGTACTCCATTCG<br>CATGGAAATCACAACAGAATTT	$(CTCTA)_4$	221	-
27	916145F<br>916145R	CAGGTCAATTACCGCGAACT<br>TTGCATCCGAAGAAAATGAA	$(GTGAAA)_3$	222	-
28	951157F<br>951157R	CGACCGTTCCTAACGTACTTG<br>GGTCAGAAGAAGGCGAACAG	$(CTT)_7$	221	-
29	951171F<br>951171R	CGCGGCTAACTTTTGTCTTT<br>ACTTCCGCCCATTCAATCTA	$(GAT)_7$	223	UP, SL<br>GAn: KT591174
30	959186F<br>959186R	GGGAAGAAGAGGAGCAGGAG<br>AACTTTCGTAGGCTGTCAAATG	$(AGA)_6$	221	-
31	1008136F<br>1008136R	TGGTTTTTAATCGATATTTCATTGTC<br>TGTTTCACGTGGTACGATTCA	$(GAAGAT)_4$	232	-
32	1028155F<br>1028155R	TTTTCTTCGCCTTCCACTTC<br>CCTGTGCACGAGAGATCCAT	$(GAA)_7$	223	-
33	1043174F<br>1043174R	GGGTGTGAGTTAGCATCAGC<br>CGTGATTCGACTGCACTTTT	$(GA)_{11}$	223	-
34	1109153F<br>1109153R	CGATCGGCTTTCGTTATTTC<br>CACTGTCACTTTCGCCCTCT	$(AGG)_7$	220	-
35	1109178F<br>1109178R	AGGGGTTTTCTTTTCGATGA<br>CGAAGGTCGGCTTGAATAAA	$(AGG)_7$	220	-
36	1158150F<br>1158150R	GCGACTATGGAAGAATTTCCTAA<br>TGTTTTCAGCCTCGTCTTCA	$(GAT)_6$	220	-
37	1171190F<br>1171190R	GCACGAAAGTCCCGAAGATA<br>TGAGGACATAGCCGTACTCG	$(ACA)_7$	222	-
38	1192175F<br>1192175R	ATCTCAATCCGATGGCTCTG<br>AAAAAGCGGTGTGGTGCTAT	$(CTGT)_6$	227	-
39	1444161F<br>1444161R	ATCTCAATCCGATGGCTCTG<br>AGATCCGCAGTTCAAACGTC	$(GTCT)_5$	220	-
40	1539174F<br>1539174R	TCGGCGAGATTTGAATTTTT<br>AGGATGTCCCACATTTGACG	$(TG)_{10}$	220	UP, SL<br>GAn: KT591175
41	1627163F<br>1627163R	CGAGATCGAGAATGAAACCAA<br>AACGAAGGCAAACGAGGAC	$(CGCGG)_4$	223	UP
42	1632148F<br>1632148R	GACGATATGTGGGCAATTCA<br>TATCTTCGACTTCGGCTTCG	$(CAA)_6$	220	-

Table S1. *Cont.*

No.	Locus	Primer Sequence	Repeated Motif	Sequence Size	Remarks
43	1657165F<br>1657165R	TTGCACGACCACAAAAATGT<br>ATCCGTTGAGAGAACGATGC	$(CAA)_6$	220	UP
44	1744180F<br>1744180R	GCCCCCAAAAGAAAAAGAAA<br>ATCATGCCATGTCCCAACTT	$(AC)_{11}$	223	UP
45	1866170F<br>1866170R	ACCATCAATTTCTGCAACCA<br>GAGCGATACCTATGCACTCTATT	$(TGTA)_5$	220	-
46	1949162F<br>1949162R	ATCTCAATCCGATGGCTCTG<br>TGTGCCACGTTTAGATTTCA	$(TGTC)_5$	221	UP
47	1958160F<br>1958160R	CACAAACCTTCCTCCACGAC<br>CGGCAGAGACAGACGTGAT	$(CCT)_7$	223	-
48	1958168F<br>1958168R	CGCTTTGGCTTCCATAACTT<br>TGCCGTCAAAGGAATATGTTT	$(ACC)_7$	221	-
49	1958179F<br>1958179R	GCCACCTCCACTGTATCCTAA<br>GGGGTGGTGGTTATGGTAATC	$(TCC)_7$	221	UP
50	2030171F<br>2030171R	AAACTACCGGTTTGCCTGTG<br>CAAGTTTGAATTCACAAAACGAA	$(TCA)_{10}$	231	-
51	2051159F<br>2051159R	CCCGTCACTGCAAATTGAT<br>AAGGCGTCGTCGTCATTAAC	$(TCC)_{12}$	237	UP
52	2051169F<br>2051169R	GTCGACCGTCATTTGTTCCT<br>CGTTTTCAGGCTCCATTCAT	$(CGA)_6$	193	UP
53	2055128F<br>2055128R	GCCGAAATTCTTTGTTGCTG<br>TGACGTTTGTTAGCGATTCAA	$(CA)_{11}$	222	-
54	2204176F<br>2204176R	ATATTCGCGAGTGACAGACG<br>AATACGTTCGCAGCAAACAA	$(AG)_{12}$	224	-
55	2204178F<br>2204178R	CGCGTCATTTAGCGAATTTT<br>AGACGCAACACAACATTTGAA	$(TGCC)_5$	220	-
56	2316127F<br>2316127R	GCAGCCTTCAAGACAGGATT<br>TTGCCTTCCTACTTCCTCCA	$(TGG)_9$	227	-
57	2441163F<br>2441163R	TTTCCAAACTCAAAATGCACTG<br>TGTAAATAAAATTAGCTCTACGTGTCA	$(ATAC)_5$	222	-
58	2538170F<br>2538170R	CGGACGAGGTTCCTGTCTAT<br>ATCTCCACGGTCATGCTTTT	$(TC)_{14}$	228	-
59	2547173F<br>2547173R	AATTGGGCCACGAAGTTTTT<br>AACAACGCCAGAAGGAGAAA	$(TC)_{14}$	200	UP
60	2715181F<br>2715181R	TATAAGGCGCACAGATTGTC<br>GACCCAATCAACTTTAACCTG	$(TGC)_9$	227	-
61	2733154F<br>2733154R	TAAACCAGTTTGTGCAATGG<br>ATGAGAGCTAATGGCTACGG	$(GGC)_7$	222	UP
62	2854160F<br>2854160R	CCGAATCCTCCTTTCCATC<br>CGTATTCAGCTGGCTACTCCA	$(CCG)_6$	220	
63	2888152F<br>2888152R	CGTGTGCAATTTTTCTTTTTC<br>CCAACTAACTCACTTCCCTAGTTT	$(TC)_{17}$	235	UP
64	2983187F<br>2983187R	GCGGCATTAAAACTCTACAGGA<br>TCACCTATCGGTATAGCCTCAAA	$(TCT)_{11}$	235	UP

Table S1. *Cont.*

No.	Locus	Primer Sequence	Repeated Motif	Sequence Size	Remarks
65	3014173F<br>3014173R	ATGTGCGACTGAAAATTCTGG<br>GAGCAGATCAAAGCCCCTATAA	$(CGA)_8$	225	UP
66	3056155F<br>3056155R	ACACAGAAGCCAACAGTAGCC<br>TATGGGAGTCACTTCGTCTGC	$(AC)_{10}$	221	-
67	3075173F<br>3075173R	CGCGGAATATAAAGTCGTGTT<br>GAAAGTAATCACCGCGCTAAA	$(GTG)_7$	221	UP
68	3217182F<br>3217182R	CCCTTCTTGTCTAGGGAGACC<br>GAGGTGAGAAGGGCTAACTCG	$(CAGA)_5$	203	-
69	3389169F<br>3389169R	GTGCCAGGAACACGATTACAC<br>GTTCAAAAGGGGTCGGATATT	$(AC)_{12}$	225	-
70	3407159F<br>3407159R	CATGCAAAAGCACGTATCACTT<br>TAGACCGAGATTTGGGGATTTA	$(GTC)_8$	224	-
71	3545149F<br>3545149R	AAATGTTGCTATTACTGAAACACAAA<br>TGTAACATCGGAACTATGTACGTTT	$(AC)_{14}$	167	-
72	3596176F<br>3596176R	ATACACGGCCAGTTCTATGATTT<br>ACTGAAAACGAACCTCTATCCAC	$(TG)_{10}$	220	UP
73	3620155F<br>3620155R	ATTGGAAATTTGAAGAGCCAAA<br>AAAACTTTAGAAGGCGTGGCTA	$(GA)_{11}$	223	-
74	3620160F<br>3620160R	TCAAGAATCAGAACATGAAAGCA<br>AAAAACCAACTCGTCATAAACCA	$(TC)_{10}$	221	-
75	3620168F<br>3620168R	GAAAAATGAGCAGAAGTTTTTCG<br>CAAGACTCGTCTTTCATCCATCT	$(AAG)_6$	219	-
76	3620171F<br>3620171R	AAAGGGGAAAAAGATTGGAGAA<br>ATTTTCCTATTGCGCTTCCTTT	$(AAG)_7$	224	-

Table S2. Collecting information and life stage of *D. hopei* individuals used for NGS and marker utility test (CRW, Capture from Rotten Woods; LT, Light Trap; WI, Wild Individual; BI, Breeding Individual).

No.	Collecting Site	Collecting Date	Life Stage	No. of Individual	Collecting Method	Remarks
1	Korea Jeollabuk-do Wanju Yongjin-myeon Hagae Maeul	5 February 2013	Adult	1	CRW	WI (Template DNA for NGS)
2	Korea Jeollabuk-do Wanju Yongjin-myeon Gueok-ri	20 December 2008<br>7 March 2015	Adult<br>Larva	8<br>12	CRW<br>CRW	WI
3	Korea Chungcheongnam-do Nonsan Yeonsan-myeon	No data	Adult	37	-	BI (emergence date: January 2013)
4	Japan Honshu Yamanashi Prefecture Nirasaki-shi	August 2012	Adult	6	LT	WI
5	China Guangxi Nanning	July 2012	Adult	17	LT	WI
6	China Shichuan Chengdu	July 2012	Adult	16	LT	WI

Table S3. Allele frequency of nine polymorphic microsatellite loci in regional populations of *Dorcus hopei*.

Microsatellite Locus	Allele Size (bp)	Guangxi China (n = 17)	Shichuan China (n = 16)	Wanju Korea (n = 20)	Nonsan Korea (n = 37)	Nirasaki Japan (n = 6)
10203	172	0.0882	0.0000	0.0250	0.0429	0.0000
10203	174	0.0000	0.0000	0.0500	0.0000	0.0000
10203	180	0.0000	0.0000	0.0750	0.0000	0.0000
10203	184	0.0000	0.0000	0.0500	0.0286	0.0000
10203	188	0.0294	0.0938	0.0000	0.2000	0.5000
10203	190	0.0882	0.1875	0.0000	0.0714	0.0000
10203	192	0.0294	0.1250	0.0000	0.0000	0.0000
10203	194	0.2059	0.1250	0.1750	0.0286	0.0000
10203	196	0.2059	0.1563	0.2750	0.1143	0.0000
10203	198	0.0882	0.2188	0.0000	0.1286	0.0000
10203	202	0.0882	0.0313	0.0750	0.0286	0.0000
10203	203	0.1176	0.0313	0.0250	0.0286	0.3000
10203	204	0.0588	0.0000	0.2500	0.3286	0.2000
10203	252	0.0000	0.0313	0.0000	0.0000	0.0000
76181	176	0.5000	0.5313	0.5000	0.5000	0.4167
76181	181	0.5000	0.4688	0.5000	0.5000	0.5833
271200	186	0.0294	0.1250	0.0000	0.0000	0.0000
271200	188	0.0294	0.0313	0.0000	0.0000	0.0000
271200	190	0.0000	0.0000	0.3000	0.1216	0.0000
271200	192	0.0000	0.0938	0.0000	0.0000	0.1000
271200	194	0.1176	0.0625	0.1000	0.0000	0.0000
271200	196	0.0294	0.0625	0.0750	0.3514	0.3000
271200	198	0.4412	0.1250	0.4750	0.4595	0.5000
271200	200	0.2941	0.4063	0.0000	0.0405	0.0000
271200	202	0.0294	0.0625	0.0000	0.0135	0.0000
271200	206	0.0000	0.0000	0.0500	0.0135	0.1000
271200	210	0.0000	0.0313	0.0000	0.0000	0.0000
271200	211	0.0294	0.0000	0.0000	0.0000	0.0000
516192	178	0.0588	0.1250	0.0000	0.1216	0.0000
516192	180	0.0294	0.0313	0.0000	0.0000	0.0000
516192	182	0.0882	0.0313	0.0000	0.0270	0.0000
516192	184	0.2941	0.4063	0.2750	0.3108	0.0000
516192	186	0.1471	0.1250	0.0000	0.0135	0.0000
516192	188	0.0000	0.0313	0.0500	0.0811	0.0000
516192	190	0.3529	0.2188	0.6750	0.4324	0.8750
516192	200	0.0000	0.0000	0.0000	0.0000	0.1250
516192	208	0.0000	0.0000	0.0000	0.0135	0.0000
516192	218	0.0000	0.0313	0.0000	0.0000	0.0000
516192	224	0.0294	0.0000	0.0000	0.0000	0.0000

Table S3. *Cont.*

Microsatellite Locus	Allele Size (bp)	Guangxi China (n = 17)	Shichuan China (n = 16)	Wanju Korea (n = 20)	Nonsan Korea (n = 37)	Nirasaki Japan (n = 6)
585164	122	0.0294	0.0625	0.0000	0.0000	0.0000
585164	160	0.5882	0.5938	0.3750	0.2973	0.1000
585164	162	0.3235	0.2813	0.2750	0.0676	0.1000
585164	164	0.0294	0.0313	0.0250	0.1892	0.4000
585164	170	0.0294	0.0000	0.0000	0.0000	0.0000
585164	174	0.0000	0.0313	0.2500	0.4459	0.4000
585164	176	0.0000	0.0000	0.0250	0.0000	0.0000
585164	208	0.0000	0.0000	0.0250	0.0000	0.0000
585164	214	0.0000	0.0000	0.0250	0.0000	0.0000
638128	171	0.0455	0.0000	0.0000	0.0000	0.0000
638128	177	0.0000	0.0000	0.0250	0.0000	0.0000
638128	179	0.0000	0.0313	0.0000	0.0000	0.0000
638128	183	0.0000	0.0938	0.0250	0.0000	0.0000
638128	219	0.0000	0.0313	0.0000	0.0000	0.0000
638128	225	0.0000	0.0000	0.0000	0.0417	0.0000
638128	227	0.7273	0.4688	0.7500	0.7917	0.6250
638128	229	0.0000	0.0000	0.0000	0.0000	0.2500
638128	230	0.0000	0.0313	0.0000	0.0000	0.0000
638128	231	0.0000	0.0000	0.0000	0.0139	0.0000
638128	243	0.0909	0.0625	0.0000	0.0000	0.0000
638128	244	0.0000	0.0000	0.0750	0.0000	0.0000
638128	245	0.0000	0.0000	0.1250	0.0556	0.0000
638128	247	0.1364	0.2500	0.0000	0.0000	0.1250
638128	251	0.0000	0.0313	0.0000	0.0972	0.0000
668183	148	0.0294	0.0333	0.0000	0.0000	0.0000
668183	160	0.0588	0.0000	0.2500	0.4722	0.6250
668183	162	0.1471	0.2000	0.0000	0.0139	0.1250
668183	164	0.5588	0.4333	0.2750	0.1528	0.0000
668183	166	0.0294	0.1000	0.0000	0.0000	0.0000
668183	168	0.0294	0.0333	0.0000	0.0000	0.0000
668183	174	0.0294	0.0333	0.0000	0.0000	0.0000
668183	176	0.0000	0.0000	0.0000	0.0278	0.0000
668183	180	0.0588	0.0000	0.3500	0.1806	0.1250
668183	182	0.0588	0.1667	0.1250	0.1528	0.1250
951171	164	0.0882	0.0333	0.1000	0.0676	0.0000
951171	166	0.0000	0.0333	0.0500	0.0000	0.0000
951171	170	0.9118	0.9333	0.8500	0.9324	1.0000

Table S3. *Cont.*

Microsatellite Locus	Allele Size (bp)	Guangxi China (n = 17)	Shichuan China (n = 16)	Wanju Korea (n = 20)	Nonsan Korea (n = 37)	Nirasaki Japan (n = 6)
1539174	164	0.0000	0.0625	0.0250	0.0286	0.0000
1539174	166	0.0625	0.0313	0.0000	0.0000	0.0000
1539174	168	0.3750	0.4375	0.1500	0.1571	0.3000
1539174	170	0.0938	0.0313	0.0250	0.0000	0.1000
1539174	172	0.1250	0.0938	0.1000	0.1143	0.0000
1539174	174	0.1250	0.0938	0.5500	0.5714	0.4000
1539174	176	0.0938	0.0938	0.0750	0.1143	0.0000
1539174	177	0.0313	0.0313	0.0750	0.0143	0.2000
1539174	178	0.0000	0.0938	0.0000	0.0000	0.0000
1539174	182	0.0625	0.0313	0.0000	0.0000	0.0000
1539174	186	0.0313	0.0000	0.0000	0.0000	0.0000
